# Analysing the trend over time of antibiotic consumption in the community: a tutorial on the detection of common change-points

**DOI:** 10.1093/jac/dkab180

**Published:** 2021-08-01

**Authors:** Robin Bruyndonckx, Samuel Coenen, Niels Adriaenssens, Ann Versporten, Dominique L Monnet, Herman Goossens, Geert Molenberghs, Klaus Weist, Niel Hens, the ESAC-Net study group, Reinhild Strauss, Eline Vandael, Stefana Sabtcheva, Marina Payerl-Pal, Isavella Kyriakidou, Jiří Vlček, Ute Wolff Sönksen, Elviira Linask, Emmi Sarvikivi, Philippe Cavalié, Tim Eckmanns, Flora Kontopidou, Mária Matuz, Gudrun Aspelund, Karen Burns, Filomena Fortinguerra, Andis Seilis, Rolanda Valintėlienė, Marcel Bruch, Peter Zarb, Stephanie Natsch, Hege Salvesen Blix, Anna Olczak-Pieńkowska, Ana Silva, Gabriel Adrian Popescu, Tomáš Tesař, Milan Čižman, Antonio López Navas, Vendela Bergfeldt, Berit Müller-Pebody

**Affiliations:** 1 Laboratory of Medical Microbiology, Vaccine & Infectious Disease Institute (VAXINFECTIO), University of Antwerp, Antwerp, Belgium; 2 Interuniversity Institute for Biostatistics and statistical Bioinformatics (I-BIOSTAT), Data Science Institute, Hasselt University, Hasselt, Belgium; 3 Centre for General Practice, Department of Family Medicine & Population Health (FAMPOP), University of Antwerp, Antwerp, Belgium; 4 Disease Programmes, European Centre for Disease Prevention and Control, Stockholm, Sweden; 5 Interuniversity Institute for Biostatistics and statistical Bioinformatics (I-BIOSTAT), Catholic University of Leuven, Leuven, Belgium; 6 Centre for Health Economic Research and Modelling Infectious Diseases, Vaccine & Infectious Disease Institute (VAXINFECTIO), University of Antwerp, Antwerp, Belgium

## Abstract

**Objectives:**

This tutorial describes and illustrates statistical methods to detect time trends possibly including abrupt changes (referred to as change-points) in the consumption of antibiotics in the community.

**Methods:**

For the period 1997–2017, data on consumption of antibacterials for systemic use (ATC group J01) in the community, aggregated at the level of the active substance, were collected using the WHO ATC/DDD methodology and expressed in DDD (ATC/DDD index 2019) per 1000 inhabitants per day. Trends over time and presence of common change-points were studied through a set of non-linear mixed models.

**Results:**

After a thorough description of the set of models used to assess the time trend and presence of common change-points herein, the methodology was applied to the consumption of antibacterials for systemic use (ATC J01) in 25 EU/European Economic Area (EEA) countries. The best fit was obtained for a model including two change-points: one in the first quarter of 2004 and one in the last quarter of 2008.

**Conclusions:**

Allowing for the inclusion of common change-points improved model fit. Individual countries investigating changes in their antibiotic consumption pattern can use this tutorial to analyse their country data.

## Introduction

The European Surveillance of Antimicrobial Consumption Network (ESAC-Net^1^, formerly ESAC) is an international network of surveillance systems that enables data on antibiotic consumption to be collected across the EU/European Economic Area (EEA). With these data, various aspects of antibiotic consumption in the community (i.e. primary care sector), including changing trends in consumption of main antibiotic groups, have been studied in a previous series of articles.^2–7^ However, while these changes could have occurred gradually, which would require a time-trend, they could also have occurred more abruptly, which would necessitate the inclusion of changes in the time-trend, referred to as change-points. Comparison of the location of these change-points with the timing of public campaigns could provide valuable insights into the effectiveness of such campaigns.

Change-points, also referred to as transition-points, switch-points or break-points, are usually estimated using either the likelihood framework^8–10^ or the Bayesian framework.^11–13^ When comparing the two approaches, the likelihood framework is computationally faster and does not require the specification of prior distributions while the Bayesian framework is less sensitive to starting values and allows the location of the change-points to be data-driven.^14^^,^^15^ For this tutorial, we focus on an adaptive Bayesian model in which both the number of common change-points and their location(s) are data-driven.^16^ This model was applied in this series of articles that reviews temporal trends, seasonal variation and presence of change-points, and composition of antibiotic consumption in the community for the period 1997–2017.^17–22^

In subsequent sections of this tutorial, the data and the application of the adaptive Bayesian model to antibiotic consumption in the EU/EEA community are presented step by step. The data structure and procedures used to fit the models are presented in Appendix 1 (available as Supplementary data at *JAC* Online).

## Data

This tutorial explains how a change-point model can be fitted to quarterly data on antibiotic consumption in the community of 25 EU/EEA countries for the period 1997–2017. Data were expressed in DDD (ATC/DDD index 2019) per 1000 inhabitants per day and aggregated at the level of the active substance, in accordance with the WHO ATC classification.^23^ The methods for collecting the data are described in the introductory article of this series.^24^ The structure of the dataset is presented in Appendix 1 (available as Supplementary data at *JAC* Online).

**Figure 1.**
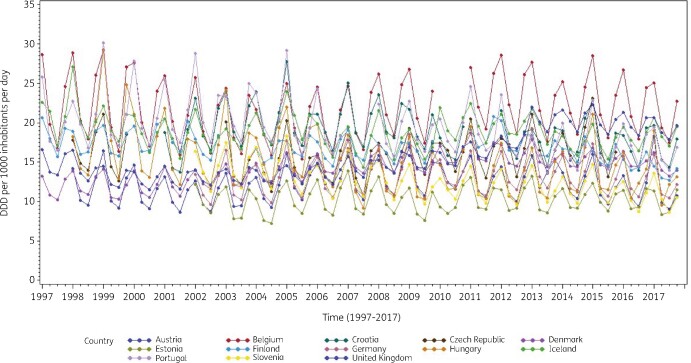
Seasonal variation in consumption of antibacterials for systemic use (J01) in the community, expressed in DDD (ATC/DDD index 2019) per 1000 inhabitants per day, in 13 countries reporting consumption per quarter for at least 15 years, 1997–2017.

Consumption of antibacterials for systemic use (ATC J01) for a subset of countries that reported quarterly data for at least 15 years during the period 1997–2017 is presented in Figure 1. These longitudinal profiles show clear seasonal variation in antibiotic consumption with upward winter peaks and downward summer troughs, associated with seasonality in viral and bacterial pathogens.^25^ In addition, the profiles demonstrate homogeneity within and heterogeneity across countries. The less complete series were for countries that did not join the network since its start, missed intermittent calls for quarterly data, or did not yet submit quarterly data for the more recent years.

## Methodology

The different steps taken in analysing quarterly antibiotic consumption data are described in subsequent sections. An illustration of how the methodology can be applied is also presented. All models are fitted in a fully Bayesian way. To ensure convergence, we recommend the use of two chains with 110 000 iterations, of which the first 10 000 iterations must be discarded, i.e. burn-in. Thinning, i.e. discarding samples, to every 5th sample is recommended because of the autocorrelation present for some of the parameters in the model. The code used to fit the models can be found in Appendix 2 (available as Supplementary data at *JAC* Online).

## Model without change-points

To model antibiotic consumption data, we needed a model that accounts for (a) homogeneity in observations within countries, (b) heterogeneity in observations between countries and (c) seasonal variation. One admissible approach is the non-linear mixed model in which random effects represent the country-specific deviations from the average and a sine wave captures the seasonality. An advantage of this model is that it does not require balanced data, which makes it a very suitable approach for modelling the combination of complete and incomplete longitudinal profiles for antibiotic consumption in the community. The non-linear mixed model is formulated as:

(Eq. 1) $$Y_{ij}=\left(\beta_{0}+b_{0i}\right)+\left(\beta_{1}+b_{1i}\right)t_{ij}+\left(\beta_{0}^{S}+b_{0i}^{S}+\beta_{1}^{S}t_{ij}\right)\sin\left(\omega t_{ij}+\delta \right)+\varepsilon_{ij},$$

where $Y_{ij}$ is the total antibiotic consumption in the community (in DDD per 1000 inhabitants per day) for country *i* (i=1,2,…,N) at timepoint $t_{ij}$ (j=1,2,…,84), time = 1 corresponds to the start of the study (first quarter of 1997), $\beta =(\beta_{0},\beta_{1},\beta_{0}^{S},\beta_{1}^{S},\omega ,\delta )$ is a vector of fixed effects where $\beta_{0}$ is the general intercept, $\beta_{1}$ is the general change in antibiotic consumption over time, $\beta_{0}^{S}$ is the general amplitude, $\beta_{1}^{S}$ is the general change in amplitude over time, ω is the frequency of the sine wave (=2π/T with T=4) and δ is the phase shift of the sine wave, $b_{i}=(b_{0i},b_{1i},b_{0i}^{S})$ is the vector of random effects where $b_{0i}$ is the country-specific deviation from the general intercept, $b_{1i}$ is the country-specific deviation from the general change in antibiotic consumption over time and $b_{0i}^{S}$ is the country-specific deviation from the general amplitude. We assume $b_{i}∼N(0,D)$ where D is a 3 × 3 diagonal covariance matrix. We assume that all $\varepsilon_{ij}$ are independent and normally distributed with mean zero and constant variance $\sigma_{\varepsilon }^{2}$.

## Model with one common change-point

Inclusion of a common change-point, which signals an abrupt change in the evolution of antibiotic consumption over time, modifies Equation 1 as follows:

(Eq. 2) $$\begin{array}{c} Y_{ij}=\left(\beta_{0}+b_{0i}\right)+\left(\beta_{1}+b_{1i}\right)t_{ij}+\left(\beta_{CP}+b_{\mathrm{CPi}}\right){\left(t_{ij}-CP\right)}_{+}\\ +\left(\beta_{0}^{S}+b_{0i}^{S}+\beta_{1}^{S}t_{ij}\right)\;\text{sin}\;\left(\omega t_{ij}+\delta \right)+\varepsilon_{ij}, \end{array}$$

where the fixed effects $\beta_{0},\beta_{1},\beta_{0}^{S},\beta_{1}^{S},\omega$ and δ, and the random effects $b_{0i},b_{1i}$ and $b_{0i}^{S}$ are defined as before, $x_{+}$=max(x,0), CP represents a common change-point, $\beta_{CP}$ is the general difference in the linear trend after versus before the change-point, $b_{\mathrm{CPi}}$ is the country-specific deviation from the general difference in the linear trend after versus before the change-point and $\varepsilon_{ij}$ is an unexplained error term. The location of this common change-point is data-driven.

## Model with additional common change-points

Inclusion of additional common change-points generalizes Equation 2 as follows:

(Eq. 3) $$\begin{array}{c} Y_{ij}=\left(\beta_{0}+b_{0i}\right)+\left(\beta_{1}+b_{1i}\right)t_{ij}+\sum_{k=1}^{K}\left(\beta_{k+1}+b_{(k+1)i}\right){\left(t_{ij}-CP_{k}\right)}_{+}\\ +\left(\beta_{0}^{S}+b_{0i}^{S}+\beta_{1}^{S}t_{ij}\right)\;\text{sin}\;\left(\omega t_{ij}+\delta \right)+\varepsilon_{ij}, \end{array}$$

Where $x_{+}$, the fixed effects $\beta_{0},\beta_{1},\beta_{0}^{S},\beta_{1}^{S},\omega$ and δ, and the random effects $b_{0i},b_{1i}$ and $b_{0i}^{S}$ are defined as before, *K* is the number of common change-points, for k=1,2,…,K, ${CP}_{k}$ represents the *k*^th^ common change-point, $\beta_{k+1}$ is the general difference in the linear trend after versus before the *k*^th^ change-point, $b_{(k+1)i}$ is the country-specific deviation from the general difference in the linear trend after versus before the *k*^th^ change-point and $\varepsilon_{ij}$ is an unexplained error term. The model with one change-point is gradually extended by including additional change-points of which the locations are again data-driven. When including more change-points than present in the data, the model will experience difficulties in obtaining convergence.

## Model selection

The Deviance Information Criterion (DIC) is typically used to select the model that explains the data best in a Bayesian setting.^26^ The DIC is a Bayesian equivalent to the Akaike Information Criterion (AIC) and is composed as the sum of the posterior expectation of the deviance ($\overset{¯}{D}$), which measures goodness of fit, and a penalty term for model complexity ($p_{D}$), which is given by the difference between the posterior expectation of the deviance ($\overset{¯}{D}$) and the deviance evaluated at the posterior mean ($D(\overset{¯}{\theta })$). Because $p_{D}$ is not invariant to reparameterization and can become negative, $p_{V}$ [which estimates the effective number of parameters in the model as Var(Deviance)/2] can be used instead. In model comparison, a smaller DIC represents a better fitting model. Convergence of the algorithm can be checked using trace plots.^27^

## Prior specification

To fit the above models in a fully Bayesian way, prior distributions need to be specified. As a reflection of our lack of knowledge about the regression coefficients, uninformative priors can be used. For the change-points, this translates to a uniform distribution over the whole time-range for the first common change-point, and a uniform distribution over the time-range following the previous change-point for additional change-points to avoid switching of change-points which would result in difficulties with model convergence. For the fixed and random effects, a normal prior with a large variance was used to reflect lack of prior knowledge. The selected priors are specified as follows:

$\beta_{0},\beta_{1},\beta_{\left(k+1\right)},\beta_{0}^{S},\beta_{1}^{S},\delta$ ∼ Normal (0, 1000), independently with *k=1, … K* and *K* the number of change-points,

$C_{1}$ ∼ Uniform (1,84),

$C_{k}$ ∼ Uniform ($C_{k-1}$,84),

$b_{0i}$ ∼ Normal(0,$\sigma_{b_{0}}^{2}$),

$b_{1i}$ ∼ Normal(0,$\sigma_{b_{1}}^{2}$),

$b_{(k+1)i}$ ∼ Normal(0,$\sigma_{b_{\left(k+1\right)}}^{2}$),

$b_{0i}^{S}$ ∼ Normal(0,$\sigma_{b_{0}^{S}}^{2}$).(Eq. 4)

For the hyperparameters, an uninformative inverse gamma distribution is used, which is specified as follows:

(Eq. 5) $$\sigma_{b_{0}}^{2},\sigma_{b_{1}}^{2},\sigma_{b_{\left(k+1\right)}}^{2},\sigma_{b_{0}^{S}}^{2},\sigma_{\varepsilon }^{2}\;∼\text{IGamma}\left(0.001,0.001\right),\text{independently},$$

where x ∼ IGamma(α,β) means that 1/xhas a Gamma distribution with mean α/β and variance $\alpha /\beta^{2}$.^28^

## Application of the methodology

We illustrate the methodology discussed above using ESAC-Net quarterly data on antibiotic consumption in the community for 25 EU/EEA countries during the period 1997–2017.

We considered the following models:

*Model 1*: Non-linear mixed model without change-points

*Model 2*: Non-linear mixed model with one unknown common change-point (C_1_)

*Model 3*: Non-linear mixed model with two unknown common change-points (C_1_ and C_2_ with C_1_ <C_2_)

*Model 4*: Non-linear mixed model with three unknown common change-points (C_1_, C_2_ and C_3_ with C_1_ <C_2_ <C_3_)

The results in Table 1 clearly indicate the need for at least one common change-point, which is reflected by the big decrease in DIC from Model 1 to Model 2. Including an additional unknown common change-point (Model 3) decreased the DIC further. Including a third common change-point (Model 4) resulted in non-convergence, indicating that a non-linear mixed model with two common change-points (Model 3) was the best fitting model for antibiotic consumption in the community. A summary of the posterior distributions for the model parameters in Models 1–3 is given in Table 1.

**Table 1.** Estimates for model fit and parameters: posterior means (standard errors)

Parameters	Model 1	Model 2	Model 3
DIC($p_{D}$)	5105.98	4877.68	4759.9
DIC($p_{V}$)	5119.56	4958.78	4864.17
$\beta_{0}$	17.784 (1.258)	17.392 (1.286)	18.046 (1.410)
$\beta_{1}$	0.001 (0.010)	0.014 (0.026)	−0.017 (0.022)
$\beta_{2}$	–	−0.009 (0.041)	0.054 (0.039)
$\beta_{3}$	–	–	−0.051 (0.050)
$C_{1}$	–	44.529 (1.278)	29.028 (1.186)
$C_{2}$	–	–	48.839 (4.168)
$\beta_{0}^{S}$	3.790 (0.345)	3.803 (0.351)	3.808 (0.342)
$\beta_{1}^{S}$	−0.012 (0.003)	−0.012 (0.002)	−0.012 (0.002)
δ	0.397 (0.017)	0.397 (0.016)	0.399 (0.015)
$\sigma_{b_{0}}^{2}$	42.970 (14.345)	41.289 (15.914)	40.711 (15.455)
$\sigma_{b_{1}}^{2}$	0.002 (0.001)	0.015 (0.007)	0.007 (0.004)
$\sigma_{b_{2}}^{2}$	–	0.035 (0.017)	0.029 (0.014)
$\sigma_{b_{3}}^{2}$	–	–	0.042 (0.025)
$\sigma_{b_{0}^{S}}^{2}$	2.543 (0.856)	2.556 (0.847)	2.572 (0.847)
$\sigma_{\varepsilon }^{2}$	2.146 (0.084)	1.794 (0.072)	1.646 (0.067)

DIC($p_{D}$): Deviance Information Criterion calculated using a penalty term for model complexity ($p_{D}$); DIC($p_{V}$): Deviance Information Criterion calculated using an estimate for the effective number of parameters in the model ($p_{V}$); *β_0_*, general intercept; *β_1_*, general change in antibiotic consumption over time; *β_2_*, general difference in the linear trend after versus before the first change-point; *β_3_*, general difference in the linear trend after versus before the second change-point; $C_{1}$, location of the first change-point; $C_{2}$, location of the second change-point; *β_0_^S^*, general amplitude; *β_1_^S^*, general change in amplitude over time; *δ*, phase shift of the sine wave; $\sigma_{b_{0}}^{2}$, random intercept variance; $\sigma_{b_{1}}^{2}$, random slope variance; $\sigma_{b_{2}}^{2}$, random difference (after versus before the first change-point) variance; $\sigma_{b_{3}}^{2}$, random difference (after versus before the second change-point) variance; $\sigma_{b_{0}^{S}}^{2}$, random amplitude change variance; $\sigma_{\varepsilon }^{2}$, residual variance.

The estimate for the first unknown change-point obtained from fitting Model 2 was 44.529 (last quarter of 2007). Allowing a second change-point, positioned the first change-point at 29.028 (first quarter of 2004) and the second change-point at 48.839 (last quarter of 2008).

**Figure 2.**
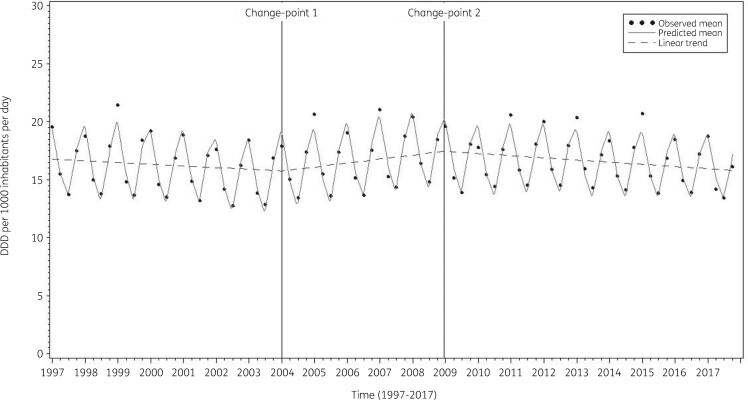
The average observed (dots), predicted (solid line) and predicted linear (dashed line) consumption of antibacterials for systemic use expressed in DDD (ATC/DDD index 2019) per 1000 inhabitants per day obtained from fitting Model 3.

**Figure 3.**
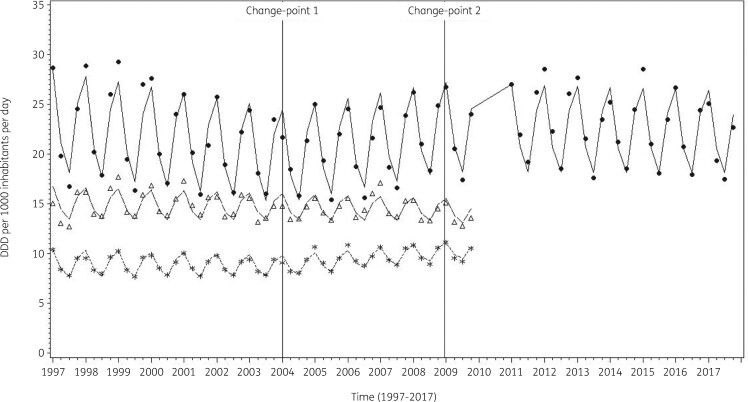
The average observed (dots, triangles and stars) and predicted (solid, dashed and dotted lines) consumption of antibacterials for systemic use expressed in DDD (ATC/DDD index 2019) per 1000 inhabitants per day obtained from fitting Model 3 for three selected countries: Belgium, Sweden and the Netherlands from top to bottom.

The average observed, predicted and predicted linear consumption of antibacterials for systemic use expressed in DDD per 1000 inhabitants per day illustrated that Model 3 fitted the data well (Figure 2). The observed and predicted consumption of antibacterials for systemic use expressed in DDD per 1000 inhabitants per day for three selected countries (Belgium, Sweden and the Netherlands) shows that the country-specific predictions closely followed the observed values (Figure 3).

## Discussion

In this tutorial, the methodology of an adaptive change-point analysis is explained and applied to quarterly data on antibiotic consumption in the community for 25 EU/EEA countries in the period 1997–2017. The inclusion of change-points in the non-linear mixed models used earlier^29^ to describe the trend and seasonal variation in antibiotic consumption over time was motivated by the need to allow for abrupt changes which then could be compared with the timing of public awareness campaigns, e.g. the European Antibiotic Awareness Day, policy changes, e.g. changing the reimbursement of antibiotics, or other interventions.^30^ This analysis can be performed at the third level of the ATC classification, as shown in the example presented here for antibacterials for systemic use (ATC J01), but could also be performed at any other ATC level, e.g. to study the evolution of consumption of amoxicillin/clavulanic acid over time. Analyses can be performed using consumption data expressed in DDD per 1000 inhabitants per day, as shown in the example presented here, but could also be performed using data expressed in any other metric of antibiotic consumption, e.g. packages or prescriptions per 1000 inhabitants per day. Analyses can be performed for multiple countries, as shown in the example presented here for 25 EU/EEA countries, but could be performed for a smaller subset of countries as well, e.g. only Northern European countries. Analyses of antibiotic consumption in one specific country could be conducted after removing the now-redundant random effects ($b_{oi},b_{1i},b_{\left(k+1\right)i}\;\mathrm{and}\;b_{0i}^{S}$) from the models. Analyses of yearly antibiotic consumption could be conducted after removing the now-redundant sine wave from the models.

While the fitted change-points were motivated by the need to account for abrupt changes following antimicrobial awareness campaigns, several other events could serve as an explanation for the observed changes in antibiotic consumption. Examples include shortages in specific compounds (e.g. narrow-spectrum penicillins in Belgium),^31^ product restrictions (e.g. EMA recommendations on fluoroquinolones),^32^ changes in package size possibly driven by companies packaging practices,^33^^,^^34^ and accordance of package size with treatment recommendations^35^ or even a pandemic (COVID-19).^36^

The models described in this tutorial could be extended by including country-specific latent indicators, thus allowing the common change-point to be switched off for individual countries. A limitation of this approach is that it hinders convergence, often limiting the model to the inclusion of one common change-point with a country-specific latent indicator. Because the model with two change-points fitted the data best, we did not consider including country-specific latent indicators as a valuable alternative in the current setting. Another option that makes the models described in this tutorial even more flexible would be to include country-specific rather than common change-points (with country-specific latent indicators). However, the added complexity often limits the number of change-points that could be included to one single change-point. Because our main interest was in the trend of antibiotic consumption over time for the EU/EEA, we preferred the inclusion of a higher number of common change-points over the inclusion of country-specific change-points. For individual countries, investigating the trend and changes in their own antibiotic consumption might be a valuable exercise when evaluating the impact of public awareness campaigns, changes in regulations and other national or international interventions.

In conclusion, allowing for the inclusion of common change-points improved model fit. Individual countries investigating changes in their antibiotic consumption patterns can use this tutorial to analyse their country data.

## Supplementary Material

dkab180_Supplementary_Data

## Supplementary data

Appendixes 1 and 2 are available as Supplementary data at *JAC* Online.
