# Supplementary material for: Analysing the trend over time of antibiotic consumption in the community: a tutorial on the detection of common change-points
Source: J Antimicrob Chemother. 2021 Aug 1;76(Suppl 2):ii79–85. doi: 10.1093/jac/dkab180 (PMC8314099; doi:10.1093/jac/dkab180)
Supplement: dkab180_Supplementary_Data [file dkab180_supplementary_data.docx]

**Supplementary data**

This supplementary material presents the structure of the dataset used in the analyse (Appendix 1) and the code used to conduct the analyses (Appendix 2).

# Appendix 1. Data structure

| Country | Year | Quarter | Time | DDD per 1000 inhabitants per day |
| --- | --- | --- | --- | --- |
| BE | 1997 | 1 | 1 | 28.075 |
| BE | 1997 | 2 | 2 | 19.789 |
| BE | 1997 | 3 | 3 | 16.722 |
| BE | 1997 | 4 | 4 | 24.579 |
| BE | 1998 | 1 | 5 | 28.249 |
| BE | … | … | … | … |
| BE | 2017 | 3 | 83 | 17.486 |
| BE | 2017 | 4 | 84 | 22.709 |

# Appendix 2. Software code

This supplementary material presents the code used to conduct the analyses. Both R (code written in R-3.4.2) and WINBUGS (code written in WINBUGS14) are needed to run the analyses reported in this tutorial.

**WINBUGS code**

The following WinBUGS code was launched from within R (see next section) to fit the change-point model with one unknown change-point.

# Model specification
Model{
for (i in 1:N){
Y[i] ~ dnorm(mu[i],tau)
mu[i] <- (B0 + b[ID[i],1]) + (B1 + b[ID[i],2])*T[i] +
 (B2 + b[ID[i],3])*(T[i]-C1)*step(T[i]-C1) +
 (alpha + b[ID[i],4] + alphaTime*T[i])*sin(1.570796*T[i] + delta)}

# Priors for random effects
for (j in 1:M){
b[j,1] ~ dnorm(0,b0.tau)
b[j,2] ~ dnorm(0,b1.tau)
b[j,3] ~ dnorm(0,b2.tau)
b[j,4] ~ dnorm(0,b3.tau)}

# Priors for fixed effects
B0 ~ dnorm(0,0.0001)
B1 ~ dnorm(0,0.0001)
B2 ~ dnorm(0,0.0001)
alpha ~ dnorm(0,0.0001)
alphaTime ~ dnorm(0,0.0001)
delta ~ dnorm(0,0.0001)
C1 ~ dunif(1,84)

#Hyper priors
tau ~ dgamma(0.001, 0.001)
b0.tau ~ dgamma(0.001, 0.001)
b1.tau ~ dgamma(0.001, 0.001)
b2.tau ~ dgamma(0.001, 0.001)
b3.tau ~ dgamma(0.001, 0.001)

#parameters to monitor
sigma <- 1/tau
sigma_b0 <- 1/b0.tau
sigma_b1 <- 1/b1.tau
sigma_b2 <- 1/b2.tau
sigma_b3 <- 1/b3.tau }

**R code**

The following R code was used to set the correct parameters for running the change-point model in WinBUGS.

require(R2WinBUGS)

# Specify location of WinBUGS on your device
BugsPath = "C:/Program Files/WinBUGS14/"

# Create the data to be used by WinBUGS

data = list(N=length(timepoints), M=length(unique(id_country)), Y=observed_DID,

ID=id_country, T=timepoints)

names(data) = list("N","M","Y","ID","T")

# List parameters for which output is needed

parameters=c("B0","sigma_b0","B1","sigma_b1",

"B2","sigma_b2","C1",

"alpha","sigma_b3","alphaTime","delta","sigma")

# Set starting values (for two chains)

inits1=list(B0=17.6,b0.tau=0.0241,B1=0.0029,b1.tau=455.8,

B2=0.5, b2.tau=0.5, C1=18,

alpha=3.45,b3.tau=0.4723,alphaTime=0.5, delta=0,tau=0.2453,

b=structure(.Data=c(rep(0,each=4,times=M)),.Dim=c(M,4)))

inits2 = list(B0=16,b0.tau=1,B1=-0.5,b1.tau=1,

B2=1,b2.tau=1, C1=65,

alpha=1, b3.tau=1,alphaTime=-1, delta=1,tau=1,

b=structure(.Data=c(rep(0,each=4,times=M)),.Dim=c(M,4)))

inits = list(inits1,inits2)

# Link to the WinBUGS file containing the model to be fitted

model = "Model.txt"

# Call the bugs function

sims = bugs(data,inits,parameters,model,n.chains=2,n.iter=110000,n.burnin=10000,

n.thin=5,digits=4,bugs.directory=BugsPath,debug=F,codaPkg=F)

print(sims,digits=3)
